# Dynamic resilience and adaptation among Chinese military veterans: risk and protective factors during transitional periods

**DOI:** 10.3389/fpsyg.2025.1671815

**Published:** 2025-12-04

**Authors:** Lina Zhao, Jingwen Bao, Xinyun Zhang

**Affiliations:** 1 School of Humanities and Social Sciences, Jiangsu University of Science and Technology, Zhenjiang, China; 2 School of Marxism, Jiangsu University of Science and Technology, Zhenjiang, China

**Keywords:** veterans, resilience, transition, adaptation, risk factors, protective factors

## Abstract

**Aim:**

This study aimed to construct a dynamic, stage-based model of resilience development among Chinese military veterans and to analyze the evolving mechanisms of risk and protective factors across phases of post-service reintegration.

**Methods:**

Thirty-seven veterans participated in the study. Using grounded theory, qualitative data from 25 in-depth interviews with Chinese veterans were coded and analyzed via NVivo 12.0 to construct stage-based resilience models. Theoretical saturation was tested with 12 additional cases, ensuring the validity of emergent categories and dynamic patterns of risk and protection.

**Results:**

The study revealed a five-phase dynamic mechanism of veterans’ resilience, encompassing Identity Separation, Adaptation Conflict, Readaptation and Reshaping, Resilience Leap, and Societal Reintegration. Each phase is marked by distinct risk trajectories, ranging from identity rupture and social detachment to cultural dissonance, role ambiguity, and fluctuations in belonging during reintegration. Protective factors evolve across these phases, shifting from relational anchors such as family and comrades to structural supports provided by institutional guidance and peer mentorship, and ultimately to an internalized sense of agency expressed through goal setting and civic responsibility. This phased structure underscores resilience as a temporally layered, identity-centered, and interaction-driven process rather than a fixed trait. The mechanism highlights transitional challenges, including unstable group belonging, emotional instability, limited access to policy resources, and difficulties in achieving social recognition during reintegration. These findings indicate that resilience development relies on cumulative interactions between risk and protection, identity fluidity, and adaptive growth, while also exposing gaps in sustained resource integration and societal validation that can compromise the stability of long-term reintegration.

**Conclusion:**

This study demonstrates that veterans’ resilience is a multi-phase, structurally evolving process shaped by shifting psychosocial risks and layered protective forces. By integrating grounded data into a stage-based model, the research provides conceptual clarity and practical guidance for targeted interventions. The findings reveal the necessity of stage-specific policy design, multi-level support integration, and sustained post-transition monitoring. This work contributes to advancing resilience theory in post-service contexts and offers a culturally relevant framework to enhance long-term social reintegration of veterans.

## 1 Introduction

Since the establishment of the People’s Republic of China, the veteran population has grown to approximately 57 million (Shi, 2025). Compared with the general adult population, veterans represent a high-risk group (Bonanno et al., 2012; Bonanno et al., 2011). A substantial body of research indicates that the transition from military to civilian life constitutes a comprehensive life-course transformation that spans structural, interpersonal, cultural, and identity-related domains (Ackerman, 1945; Hunter-Johnson et al., 2020). This shift involves profound realignments of daily routines, support networks, modes of participation, value systems, life purposes, role identities, lifestyle patterns, skill sets, and self-concept. Collectively, these elements make this transition one of the most significant and challenging transformations an individual can undergo (Ackerman, 1945; Mobbs and Bonanno, 2018; Saleem et al., 2020). Empirical studies further demonstrate that many veterans experience considerable psychosocial stressors during the reintegration process, which often manifest as psychological distress, emotional instability, and behavioral conflicts (McDaniel et al., 2024). Simultaneously, the erosion of military culture, the disruption of self-identity, and the loss of life goals frequently trigger acute psychological and emotional dissonance, as well as maladaptive behavioral patterns (Romaniuk et al., 2024; Cramm et al., 2020). In the Chinese context, researchers have identified several pressing challenges associated with veterans’ reintegration. Veterans commonly face gaps between their expectations for social security and the actual availability of benefits, alongside heightened job insecurity, economic hardship, and a lack of transferable skills. These issues collectively exacerbate their vulnerability to financial marginalization and social exclusion (Hu and Wu, 2021). Furthermore, many veterans report feeling a lack of societal recognition for their service contributions, as well as inconsistencies in policy implementation across localities (Diamant and O’Brien, 2015; Yang, 2024). These interconnected challenges diminish veterans’ sense of belonging and social integration. In certain cases, they contribute to collective grievances or adverse social behaviors, which in turn present broader risks to social cohesion (Hu and Wu, 2021).

The transition from military service to civilian life exposes veterans to a spectrum of psychosocial stressors while simultaneously activating their intrinsic resilience (King et al., 1999). Resilience is conceptualized as an individual’s capacity to initiate adaptive cognitive, emotional, and behavioral processes in response to external challenges, thus facilitating recovery and growth in the face of adversity (Ungar, 2018). In combination with related protective constructs such as gratitude, a strong sense of purpose, and social connectedness, resilience serves as a critical protective mechanism. It mitigates the onset of mental health disorders and reduces physical health risks associated with the disruptions of military-to-civilian transition. Empirical evidence indicates that veterans with higher resilience levels demonstrate more effective recovery from substance use difficulties, superior management of deployment-related traumas and life course disruptions, and enhanced capacity to cope with transition-related demands. Collectively, these adaptive outcomes promote sustained psychological wellbeing and contribute to long-term health stability (Fogle et al., 2020).

Resilience involves two fundamental components, namely the exposure to risk or adversity and the subsequent demonstration of positive adaptation or competence in response to these adverse conditions (Luthar and Cushing, 1999). It is further conceptualized as an equilibrium achieved between individual strengths or protective factors and vulnerabilities or risk factors following experiences of adversity or traumatic events (Eshel and Kimhi, 2016). Risk factors refer to various influences that increase the probability of initial onset, progression toward greater severity, or the persistence of maladaptive states, as described by Kirby and Fraser (1997). These factors originate from discrete traumatic incidents, multiple concurrent life stressors, or the cumulative burden posed by personal and environmental pressures, as outlined by Tusaie and Dyer (2004). In contrast, protective factors reduce the likelihood of engaging in problematic behaviors through direct personal or social control mechanisms, such as strong religious commitment or consistent parental sanctions, through encouraging participation in activities inherently incompatible with risky behaviors, including organized family or church group involvement, and through fostering commitments to conventional social institutions, such as educational systems or adult societal roles (Jessor et al., 1995). Protective influences, therefore, can be categorized into individual dimensions, including courageous coping strategies, hope, and spiritual perspectives; family dimensions, such as supportive family atmospheres and available familial resources; and social dimensions, exemplified by accessible health services and integration within social network (Ahern et al., 2006). Collectively, these conceptual frameworks emphasize that resilience arises from the dynamic interaction between adversity exposure and the activation of protective mechanisms, thereby highlighting the necessity for an integrated examination of vulnerability and strength within empirical resilience research.

Resilience is increasingly conceptualized not as a fixed trait but as a dynamic, stage-dependent process that captures the capacity of systems or individuals to absorb disturbances, adapt to evolving conditions, and ultimately transform into more robust configurations. At the systemic level, resilience comprises a resistance phase in which systems withstand shocks while maintaining core functions, followed by an adaptation phase characterized by reorganization in response to perturbations, and subsequently a transformation phase in which fundamental structural or functional shifts establish new equilibria (Bousquet et al., 2016; Freitas and Downey, 1998). Parallel scholarship has demonstrated that individual resilience similarly unfolds through distinct temporal stages, including initial disruption, adaptation, recovery, growth, and reintegration, with each stage marking shifts in coping strategies and developmental trajectories (Conz and Magnani, 2020). Critically, risk and protective factors are neither static nor uniform. Their composition, salience, and impact evolve across these stages, thus requiring continual cultivation of protective assets and strategic mitigation of vulnerabilities (de Terte et al., 2014). Within this framework, risk factors are defined as situational or dispositional characteristics that heighten susceptibility to adverse outcomes, and their influence varies depending on timing and contextual exposure. In contrast, protective factors, such as social support networks, self-efficacy beliefs, and inherent physiological robustness, can be systematically developed and reinforced over time, reflecting the complex interplay between biological maturation and environmental resources (Vella and Pai, 2019). Given that individual resilience involves cognitive restructuring, active adaptation, and proactive coping strategies, the resilience of veterans is similarly conceptualized as a dynamic and multi-phase process (Ungar and Theron, 2020). Veterans have indicated that their experiences of resilience involve adaptability when confronted with adversity, and such adaptability develops progressively over time. This developmental trajectory is responsive to psychological and social support interventions, indicating that resilience is not merely a static trait but can be fostered through deliberate practices and modifications of the environment (Merali, 2023).

Effective support for military veterans’ transitions must be grounded in an understanding of the dynamic characteristics of resilience, accompanied by the implementation of appropriately tailored measures. By clearly identifying differentiated structural features of resilience factors and effectively leveraging the inherent dynamism within resilience processes, support initiatives can be strategically designed to facilitate veterans’ smooth reintegration into civilian contexts. Although substantial research has examined veteran resilience, studies that investigate stage-specific attributes of resilience and corresponding support mechanisms designed according to these temporal characteristics remain sparse. Particularly, systematic analyses of the differentiated interplay between risk and protective factors across distinct resilience phases are lacking.

In the Chinese context, empirical investigation into the resilience processes of veterans has not yet begun. Scholars have identified various transitional challenges confronting military veterans, and the State has established a dedicated Ministry of Veterans Affairs and issued policy documents such as the “*Opinions on Promoting Employment and Entrepreneurship for Veterans in the New Era*” (《关于促进新时代退役军人就业创业工作的意见》), the “*Guiding Opinions on Comprehensively Improving the Education and Training Work for Demobilized Soldiers*” (《关于全面做好退役士兵教育培训工作的指导意见》), and the “*Work Standards for Assisting and Supporting Veterans in Difficulty*” (《困难退役军人帮扶援助工作规范》) to facilitate their transitions. Nevertheless, the conceptualization and practical operationalization of resilience remain absent from current academic discourse and policy implementation. As a result, existing policy frameworks risk adopting a uniform approach rather than providing interventions aligned with veterans’ evolving resilience trajectories. Such gaps restrict the practical effectiveness of current support mechanisms and impede the optimal implementation of relevant policies.

## 2 Materials and methods

### 2.1 Research method

This study used grounded theory to systematically analyze extensive qualitative data, delineate veterans’ evolving resilience stages, and identify the distinct risk and protective factors associated with each phase. As a rigorous qualitative methodology, grounded theory generates multifaceted conceptual frameworks directly from systematic data collection and iterative analysis (Pan et al., 2023). Unlike deductive approaches, it forgoes predetermined hypotheses and instead relies on an inductive procedure that continuously compares new data with emerging concepts. In practice, grounded theory is particularly well suited to complex social phenomena because it keeps researchers receptive to unanticipated patterns in participants’ narratives and interactions, allowing theories to remain deeply rooted in empirical materials and responsive to lived complexity.

In resilience research, grounded theory demonstrates particular effectiveness by conceptualizing resilience as a dynamic process influenced by continuous interactions between encountered challenges and available resources, as highlighted by Nilakant et al. (2014). By enabling concepts derived inductively from veterans’ own accounts to surface, this approach provides a detailed depiction of individuals’ adaptive strategies, emotional regulation, and resource mobilization in response to adversity. Furthermore, grounded theory fosters a richly contextualized understanding of coping mechanisms and elucidates the synergistic interactions between risk exposure and protective supports within specific cultural and institutional contexts, as discussed by Ilias et al. (2019). Consequently, grounded theory offers significant theoretical depth and practical applicability, providing scholars and practitioners with a comprehensive framework for examining and enhancing resilience trajectories among diverse veteran populations.

This study specifically adopted the Straussian variant to inductively derive a staged model of veterans’ resilience while maintaining analytic rigor through constant comparison, theoretical sampling, and memoing (Corbin and Strauss, 2014). Following the initial–axial–selective coding sequence, we first conducted initial (open) coding to fracture transcripts line by line, naming incidents and specifying properties and dimensions; we then undertook axial coding to reassemble categories using the Straussian paradigm, thereby linking phase specific risks and protective processes into coherent explanatory relations; finally, selective coding integrated all major categories around a core category that captures the dynamic, multi-phase trajectory of resilience and its identity mechanisms.

### 2.2 Participants

Participants in this study were recruited through purposive sampling and snowball sampling implemented via the researchers’ social networks. These recruitment strategies were employed to facilitate the establishment of rapport and trust, thereby enabling participants to disclose comprehensive and candid accounts of their personal circumstances and to convey authentic emotional responses. The demographic and background characteristics of the participants are provided in Table 1.

**TABLE 1:** Characteristics of the study participants.

Number	Gender	Military status at discharge	Region	Age at discharge	Time since demobilization
C1	Male	Officer	Jiang Su	38	2021
C 2	Male	Soldier	Xi Zang	20	2010
C 3	Male	Non-commissioned Officer (NCO)	Si Chuan	28	2019
C 4	Male	Soldier	An Hui	22	2012
C 5	Male	Soldier	Jiang Su	26	1983
C 6	Male	Soldier	Jiang Su	22	2004
C 7	Male	NCO	Si Chuan	32	2017
C 8	Male	NCO	Si Chuan	30	2017
C 9	Male	Soldier	Jiang Su	22	2012
C 10	male	soldier	Xi Zang	24	2003
C 11	Male	NCO	Jiang Su	30	2016
C 12	Female	Soldier	Jiang Su	22	2020
C 13	Male	Soldier	Jiang Su	21	1997
C 14	Male	Soldier	Jiang Su	21	2022
C 15	Male	Soldier	Jiang Su	22	2023
C 16	Male	Soldier	Jiang Su	22	2023
C 17	Male	Soldier	Jiang Su	21	2020
C 18	Male	Soldier	Jiang Su	21	2021
C 19	Male	Soldier	Jiang Su	21	2021
C 20	male	soldier	Jiang Su	22	2022
C 21	Male	Soldier	Hei Longjiang	21	2022
C 22	Male	Soldier	Hei Longjiang	20	2021
C 23	Female	Soldier	Yun Nan	21	2021
C 24	Male	Soldier	Jiang Su	21	2021
C 25	Male	Soldier	Jiang Su	21	2023
C 26	Male	NCO	Jiang Su	25	2004
C 27	Male	Soldier	Jiang Su	21	2022
C 28	Male	Soldier	Jiang Su	21	2021
C 29	Male	Soldier	Jiang Su	26	1984
C 30	Male	Soldier	Jiang Su	27	1976
C 31	Male	Soldier	Jiang Su	20	1980
C 32	Male	Officer	He Nan	42	1994
C 33	Male	Soldier	Shang Hai	24	2019
C 34	Male	Soldier	Hai Nan	25	2010
C 35	Male	Soldier	Bei Jing	25	2011
C 36	Male	Soldier	Guang Xi	24	2022
C 37	Male	Soldier	Shen Zhen	21	2014

### 2.3 Data collection and processing

All data were collected by the full author team through face to face, semi-structured interviews using open-ended questions. The interview protocol was carefully developed to align with the research objectives and with a particular focus on capturing dynamic changes in adaptive capacity. Open-ended prompts were designed to elicit detailed, narrative-style accounts of participants’ personal experiences. To ensure clarity, relevance, and logical flow, the guide was piloted with three veteran participants. Their feedback informed targeted refinements to the wording and sequencing of questions. After these revisions, formal interviews were carried out, during which respondents described the pivotal events and transitional experiences that shaped their resilience trajectories. The specific contents explored in these interviews are summarized in Table 2.

**TABLE 2:** Interview guidelines.

Number	Contents
1	What kinds of changes did you experience when transitioning from the military back to civilian life (e.g., lifestyle habits, ways of interacting with others, rules you follow, etc.)? Were these changes stressful for you? What strategies did you use to cope, and how effective were they?
2	How would you divide your social adaptation process stage after military discharge?
3	During the adaptation process, who or what organizations provided you with help and support in adapting to your new life and environment? What specific forms of help and support did they offer? (e.g., family, friends, fellow veterans, community, schools, veterans affairs departments, the internet, etc.)
4	During the adaptation process, what factors acted as obstacles? (e.g., physical and mental health, family, environment, knowledge and skills, personality, etc.)
5	In your opinion, what was the biggest challenge you faced when returning from the military to civilian life? How did you deal with it?
6	How do people around you describe your personality? How would you describe yourself? How would you assess your abilities in interpersonal relationships, problem-solving, emotional regulation, and goal setting?
7	Overall, what do you think are the key enabling factors that help veterans adapt to civilian life and cope with adversity after discharge? What are the major barriers?

All volunteers received a comprehensive briefing about the study and were provided with 6 days to reflect before taking part in the interviews. They retained the right to withdraw at any point without consequence. To collect rich and contextually grounded data, in-depth interviews were conducted in quiet and standardized settings, most commonly in meeting rooms. During these sessions, researchers produced detailed handwritten notes and simultaneously recorded high-quality audio. Each interview lasted between 60 and 120 min and concluded when participants ceased to provide new insights, which indicated thematic saturation. All audio recordings were transcribed verbatim and stored securely in compliance with institutional data protection requirements. Transcripts from 37 interviews that met the inclusion criteria were analyzed thematically using NVivo 12.0. Of these, 25 were randomly selected for initial coding, and the remaining 12 were used to confirm data saturation and strengthen the reliability of the emergent coding framework.

## 3 Results

### 3.1 Open and axial coding process for veterans’ resilience stages

Following a rigorous process of qualitative data analysis, 168 statements were selected for open coding after multiple rounds of careful screening and verification to ensure both representativeness and analytical relevance. The research team carried out iterative, line by line coding to extract the core meanings and recurring patterns embedded within the raw data. Through continuous comparative analysis and refinement, semantically similar and thematically convergent codes were systematically consolidated, resulting in a total of 102 distinct initial categories that capture the nuanced characteristics of veterans’ resilience development. In the subsequent axial coding phase, these initial categories were further conceptualized and organized into coherent sub-categories that correspond with the dynamic stages of resilience progression. The hierarchical structure linking the main categories, sub-categories, and the corresponding initial categories is systematically presented in Table 3.

**TABLE 3:** Open and axial coding of resilience stages.

Main categories	Sub-categories	Initial concepts	Original sentences
Identity Separation	Disruption of behavior and rules	Military routine inertia	When I first got back, I was still on the military schedule, awake around six, but anyone else was still sleeping at that time. (case 22)
		Behavioral disruption	Back at school, it feels like no one’s in charge, totally different from the army. (case 21)
		Regulatory barriers and complex reenrollment procedures	I had to go through a bunch of departments, and it was incredibly cumbersome. (case 24)
	Disconnection from groups and relations	Breakdown of comrade networks	After suddenly losing contact with my comrades, even being connected on WeChat no longer feels as warm as before. (case 23)
		Estrangement from former classmates	After I came back, most of my former classmates had already graduated, and I couldn’t even recognize some of them. (case 23)
		Loss of belonging and emotional vacuum	I feel like I don’t belong anywhere. There’s no sense of belonging at home, and I can’t go back to the army. (case 6)
		Social information gap	I haven’t paid much attention to what’s been happening outside for more than two years, so I’m unaware of some of the new policies. (case 9)
	Ambiguity in roles and identity	Identity confusion	“For a time I couldn’t tell if I was a student or a soldier, I felt kind of unmoored.” (case 18)
		Temporary aimlessness	I suddenly felt I had no goals, and during the period after I was discharged I didn’t want to do anything. (case 25)
		Self-doubt about adaptability	I’m constantly worried about whether I can keep up with the pace at school. (case 25)
	Disparity in living and economics	Inadaptation to dormitory	After moving to a new dormitory, I didn’t know anyone and was too timid to talk at night. (case 16)
		Maladaptive spending habits	I spent little in the army, after coming back my expenses suddenly went up, and it feels a bit hard to adapt. (case 15)
		Conflicts in hygiene practices	I enjoy organizing, but my roommate is very messy, which I find hard to tolerate. (case 22)
		Age gap	Since I just came back and I’m older than they are, I really feel a clear generation gap when I talk with my roommates. (case 22)
	Barriers to resources and policies	Policy service disconnect	There are hardly any services at the university specifically to interface with veterans. (case 21)
		Lack of support resources and inability to seek help	When I’m confused, I don’t know who to turn to for help. (case 16)
Adaptation Conflict	Social and group alienation	Lack of belonging	I find it hard to fully fit in with my classmates; I usually prefer to spend time with my veteran friends. (case 23)
		Low motivation for proactive socialization	I’ve become more withdrawn and rarely take the initiative to chat with my classmates. (case 22)
	Lifestyle and habit conflicts	Discrepant paces of life and circadian rhythm mismatch	I’m often teased that others can sleep late, but I still wake up on the military schedule. (case 14)
		Differing consumption values	I’m quite thrifty and dislike seeing others spend extravagantly when they shop. (case 19)
		Conflicts in hygiene habits	Since I got back, the constant mess at home really bothers me, and we sometimes get into little arguments about cleaning. (case 1)
	Information and resource barriers	Complicated subsidy application	The subsidy procedures are opaque, and it took me several inquiries to finally understand them. (case 24)
		Information asymmetry and unfamiliarity with policy procedures	Some policy information comes out very slowly, and I always find out after the fact. (case 21)
		Low resource utilization	There are all kinds of activities at school, but I’m not really sure how to sign up for them. (case 23)
	Family and emotional gap	Family’s lack of understanding	My family see military service as just “training,” but I feel I’ve changed so much I can’t even put it into words. (25)
		Irritability	Since returning, I’ve become more short-tempered and have occasional conflicts with classmates. (case 18)
		Conflict in self-identity	I keep feeling people think I’m an outsider, and I’m still not really comfortable with my identity. (case 18)
	Cultural and environmental conflict	Difficulty adapting to local culture and challenge in transferring military experience	I cannot understand the local dialect, and communication with local residents is difficult. (case 17)
		Inapplicability of military experience	The stuff from the army doesn’t work the same in the local (civilian) world; I have to learn the rules all over again. (case 24)
		Weak sense of independence	In the unit, everyone had a clear division of labor. After returning to school, I have to handle everything by myself, which is very stressful. (case 21)
Readaptation and Reshaping	Social belonging and identity	Participation in social organizations	I joined a volunteer team, made lots of new friends, and felt a real sense of accomplishment. (case 12)
		Enhanced sense of belonging	I feel I’m gradually becoming part of this place. (case 16)
		Multiple identity integration	Now I feel that I am both a soldier and a student, and even a volunteer. (case 18)
	New circles and support system	Help from new friends and peer support	My classmates proactively show me around the campus and guide me through all the procedures. (case 25)
		Deeper family understanding	My family supports my plan to pursue graduate studies and is willing to handle my living arrangements. (case 16)
		Proactive guidance from teachers	The teacher often asks how I’m adapting and says I can come to the office anytime if I have any difficulties. (case 17)
	Rebuilding competence and skills	Improved hands-on skills	After returning from the army, I gradually found that my repair skills came in handy in daily life. People trust me to fix lights and appliances, and my maintenance skills have improved as well. (case 16)
		Learning new social practices	I’ve learned how to create WeChat groups and register online. I’ve picked up a lot from the younger folks. (case 16)
		New lifestyle habits	I’ve gotten used to my classmates’ routine and don’t get up early to clean anymore. (case 22)
	Emotional and psychological recovery	Emotional stabilization	When I had just come back, I tended to be irritable; now I can calm down and solve problems. (case 18)
		Regained self-confidence	When I take new classmates to activities, I introduce myself proactively and feel I’m becoming more and more confident. (case 25)
		Proactive organization of activities	I now take the initiative to organize group discussions or class dinners. (case 16)
	Resource alignment and policy utilization	Smooth policy connection	Our counselor helped us navigate the policies. There was guidance available on subsidies and even extra points for the postgraduate entrance exam. (case 21)
		Group information dissemination and enhanced information access	The staff set up a group for us and regularly post job openings and training information. (case 16)
	Behavioral habit reshaping	Formation of new routines and development of new daily habits	I gradually got used to my classmates’ schedule and no longer get up early to do the cleaning. (case 22)
Resilience Leap	Competency enhancement and output	Team leadership and practical skills improved	Having led a team in the “Sanxiaxiang” rural service initiative, I observed a rapid improvement in my coordination and organizational capacities. (case 12)
		Enhanced learning ability	I’ve been actively seeking out the teacher with questions and I’m more eager to learn than I used to be. (case 16)
		Accumulation of experience	After every activity, we review with the team to sum up lessons and improve next time. (case 23)
		Giving back capabilities	After learning e-commerce, I experimented with livestream commerce to boost our village’s economy and trained local residents to engage in online business. (case 3)
	Goal orientation and autonomy	Explicit goal-setting and goal orientation	I’ve set new goals for myself, like grad school and starting a business, and I feel busy in a good way every day. (case 20)
		proactive future planning and self-direction	I did my own research on start-up policies and planned my next steps. (case 11)
	Social responsibility and service	Heightened sense of service and join voluntary activities	I signed up for volunteer service because I felt I could do something for others. (case 10)
		Pluralistic social participation and social responsibility	Taking part in community service and innovation/entrepreneurship competitions feels really meaningful. (case 12)
		Helping new members	When a new colleague joins, I proactively share my work experience without holding anything back. (case 13)
	Maturity of mindset and self-confidence	Improved self-confidence	As I’ve grown into my veteran identity, I’ve become more confident and can confront pressure head-on. (case 21)
		Mature emotion management	When under pressure, I’m able to self-regulate proactively and seldom lose emotional control. (case 25)
		Honor-driven motivation	Recognition from the local authorities motivates me even more. (case 17)
		Positive mindset	I no longer panic when facing difficulties, and my mindset is much more positive. (case 3)
		Enduring military spirit	The toughness and resolve shaped by my service have become my defining tone. (case 24)
	Resource integration and innovation	Effective utilization of social resources,	I have gradually learned to leverage policy instruments, alumni ties, and personal networks to address challenges. (case 21)
		Interdisciplinary cooperation	I can proactively make friends with classmates outside my major and with people in the wider community. (case 12)
		Innovation and entrepreneurship	I’m running a farm, growing strawberries and tomatoes, selling agricultural products, and doing some agritourism. I chose self-employment and now I’m essentially working for myself. (case 11)
		Reflective practice	Think more, observe more, study more, and listen more, learn from others’ experience and make it my own. (case 4)
	Optimization of lifestyle	Healthy routine	I’ve consistently kept up my exercise routine, and my schedule is very regular. (case 16)
		Frugality and self-discipline and improved life skills	I have kept the self-discipline and frugality instilled by the military, and my lifestyle remains healthy. (case 22)
Societal Reintegration	Integration of multiple identities	Flexibility in identity switching and identity integration	I am now a student and a veteran, as well as a volunteer and an entrepreneur, and I can switch smoothly between these roles. (case 18)
		Advancement in family roles	I have become the role model in my family, and my younger brother and sister have begun to follow my example. (case 25)
	Social recognition and contribution	Gaining social respect	By participating in community service, I have earned recognition and respect from many people. (case 12)
		Deeper social identity	I got the veteran’ benefits card, I can use priority lines at attractions and hospitals. It makes me feel seen. (case 23)
		Increased sense of contribution	It feels truly worthwhile to turn my own experience into something the group can use. (case 7)
		Satisfaction and wellbeing	I’m very happy now, my work, life, and family are all going well. (case 22)
	Giving back and motivation	Guiding new members,	When new recruits come back, I take the initiative to show them how to adapt to their new life. (case 23)
		Sharing experience	I often answer questions in our veterans’ group chat these days. (case 16)
		Policy advocacy	I now actively participate in demobilization-policy briefings to help more fellow veterans adapt to society. (case 21)
		Positive encouragement for new members	Frequently encourage newly discharged service members to take an active part in collective activities. (case 10)
	Normalization of duty and service	Regular engagement in social service and voluntary service	Volunteer service and community public-welfare activities have become part of my life. (case 12)
		Active participation in community building	Serving as a village official in my home village, I find it deeply meaningful to contribute to my hometown. (case 23)
		Innovation and entrepreneurship	We’re a city-level model base for veterans’ entrepreneurship. Every year I go to the district Party School to offer guidance and give classes to veterans. (case 22)
	Expansion of circles and competency	Broadening social networks,	Participate in community volunteering and social activities to build new connections and relationships. (case 12)
		Mentoring capabilities	Because many local residents either do not understand Mandarin or cannot read Chinese characters, we ethnic minority cadres are needed to serve as intermediaries and play a bridging role. (case 10)
		Continued education and development	Getting into grad school made me realize this path is a great fit for me. (case 16)
		Experience-sharing with new groups	Some veterans want to start a business but feel they lack the necessary abilities. In such cases, we tailor and design a set of training methods specifically for that veteran based on the actual situation; we call this “entrepreneurship training. (case 13)
	Maturation of lifestyle	Internalized self-discipline and frugality	Over the years since returning to civilian life, I’ve continued to keep the self-discipline and frugal habits instilled in me by the army. (case 22)
		Healthy lifestyle awareness	Now I just want to nurse my health. I sing and exercise in my spare time, and I feel quite optimistic, full of strength, and resilient. (case 5)
		Overall happiness	This job has given me a real sense of purpose. Now when I walk through the village, everyone knows me. I feel truly at ease. I’m not just the village director, I’m part of the village. This is my home. (case 3)

### 3.2 Open and axial coding of risk and protective factors across resilience stages

A total of 514 discrete statements were analyzed through open coding. The research team conducted multiple rounds of iterative comparison, categorization, and theoretical sensitivity refinement, systematically synthesizing semantically and conceptually similar units. This process resulted in 236 distinct initial categories. The procedure preserved the richness of the data while improving the conceptual clarity and consistency necessary for grounded theory development. Axial coding was then applied to further organize and refine these initial categories by establishing logical linkages and causal relationships among them. This stage-specific integration provided a dynamic understanding of how diverse micro-level experiences aligned with the broader trajectory of resilience formation. The resulting primary categories and their associated initial categories are presented in Table 4, which illustrates the layered complexity and stage-sensitive characteristics of the resilience mechanisms identified.

**TABLE 4:** Open and axial coding of risk factors and protective factors for each resilience stage.

Factor type	Main categories	Initial concept	Original sentences
**Stage 1: Identity Separation**			
Risk factor	Emotional and stress fluctuations	Emotional loneliness	When I suddenly lost contact with my comrades in arms, I felt lost and unsettled. (case 23)
		Psychological stress	The change of demobilizing and returning to my local community has put quite a lot of pressure on me. (case 25)
		Anxiety about the future	Suddenly, my goals don’t feel so clear anymore. (case 7)
		Doubts about ability	I think the biggest challenge is studying. (case 25)
	Economic adaptation difficulties	Career uncertainty	When I was about to be discharged, just thinking about what I could do back home got me all worked up. I didn’t know what kind of job I could do. (case 9)
		Consumption troubles	Some people feel lost after leaving the military and may idle away their time or spend money without restraint. (case 17)
		Living expenses pressure	In the military, everything’s covered and you don’t need to spend a cent. But after you get out, you’ve got to take care of everything yourself. (case 12)
	Skills transfer dilemma	Military skills not applicable	The skills learned in the army are all military skills, which are basically not usable in study or work. (case 21)
		Insufficient social adaptation skills	Because student veterans differ from their non-veteran peers in interpersonal relationships, lifestyle habits, and study behaviors, they are prone to experiencing anxiety. (case 17)
	Identity and belonging impact	Disconnection from peer groups	Although I keep in touch with my comrades on the phone, it’s far less cordial than before. (case 23)
		Gap in military identity	When I first came back, the pace of life felt quite different. In the army everything was very regimented, but after returning to my hometown it all seemed a bit loose. (case 7)
		Ambiguous identity and self-doubt	After coming back, I also had some trouble adjusting and felt a bit lost; for a period of time I felt disconnected from that society. (case 18)
	Social and environmental adaptation obstacles	Peer barriers	I feel like someone who suddenly got inserted into the class. It really takes quite a bit of effort, and I don’t get along very well with my classmates. (case 22)
		Age differences	I’m 2 years older than they are, so our routines and how we communicate are different. (case 16)
		Conflict of living habits and difficulty in adapting to routines	My routine probably doesn’t line up with my roommate’s, and our cleanliness habits are pretty different too. (case 22)
	Social integration resistance	Lack of community support	Does the community organize such activities? Not that I’ve heard. (case 21)
		Disconnection in policy services	No clear guidance is provided. (case 17)
	Institutional and resource barriers	Complicated administrative procedures	The procedures are cumbersome, and the timeline is quite drawn out. (case 20)
		Poor communication	Sometimes it’s right at the start of term and there aren’t any teachers around—we end up waiting a long time. (case 21)
		Transition obstacles	We need to liaise with the Veterans Affairs Bureau and the relevant government departments, and some students may also need to liaise with the local community and university. (case 18)
		Gaps in policy services	Some policies are well designed, but they don’t work in practice… The rules don’t seem to be interoperable across departments such as the schools/colleges and the People’s Armed Forces Office. (case 21)
Protective factor	Personal will and quality	Strong willpower	Personality-wise, anyway, when things come up I feel I won’t give up easily, and I can overcome difficulties. (case 24))
		Emotional self-regulation and proactive adjustment	There are bound to be conflicts after demobilization; if you don’t work through them yourself, what can you do? (case 25)
		Goal orientation	I’m someone who tends to have clear goals; I begin by setting a goal for myself. (case 3)
		Character growth	Two years of military service made me much more outgoing and greatly benefited my personal development. The qualities I forged as a veteran have always sustained me. (case 23)
	Life skills reserve	Improved physical fitness	During my service in the army, my physical fitness improved significantly, which has been a favorable supporting condition after returning to the local community. (case 19)
		Enhanced life skills	In the service, we fixed the lights and plumbing ourselves. (case 16))
	Family and emotional Support	Family encouragement	My family has given me a lot of encouragement and help. (case 25)
		Family trust	They feel pretty assured about me, and I’m also a very independent person. (case 24)
		Parental acceptance	Secondly, parents and the family need to provide a certain amount of support; if that support is absent, it can actually be very harmful to veterans. (case 1)
		Inancial support from family	It’s still my family that has given more support… Overall, it’s the family that has offered more care and also some financial support. (case 11)
	Comrade support	Spiritual support from comrades	A comrade’s words gave me moral support. (case 25)
		Deep comrade friendship	My greatest gain was a very strong sense of camaraderie… We went through an unforgettable period together. Thinking of this makes me feel that difficulties can be overcome. (case 24)
		Experience sharing	One of my comrades passed on some experience to me. (case 11)
		Mutual assistance	With former comrades, we used to talk a lot while serving; after returning to our local communities, we keep communicating and exchanging with each other. (case 4)
	Campus resource empowerment	Patient guidance from teachers	The teachers are very patient, and communication among classmates is pretty good. I also keep after the teachers to ask about problems I don’t understand. (case 16)
		Senior student assistance,	A veteran who had been discharged before me rode his e-bike and took me here and there; although he was a stranger, he made me feel very warm. (case 15)
		School support	The school gave me some help… giving me the opportunity to return to school to study. (case 16)
		Exclusive policy subsidies	The school’s various policies supporting student veterans are quite touching. (case 21)
	Peer acceptance and belonging	Support from veteran student groups	The process mainly relies on what other classmates who have returned from military service share. (case 16)
		Enthusiastic help from classmates	Classmates provide assistance with daily life. (case 17))
	Institutional and social support	Veterans affairs service	The Veterans Affairs Bureau … if you have any questions, you can consult them. (case 1)
		Social welfare guarantee	They provide subsidies for wounded or disabled veterans … and will help them find suitable jobs. (case 6)
		Job advantages	If you have special-operations experience, you don’t need to take the written exam to apply for the special police (SWAT); you can go straight to the interview. (case 13)
		Professional training	The state carries out training programs in many disciplines and categories to help with employment or arrange job placements. (case 21)
**Stage 2: Adaptation Conflict**			
Risk factor	Role and identity conflict	Maladaptation to role transformation	To fit in, I need to set aside my former identity as a soldier and accept society’s standards. (case 17)
		Ambiguous role positioning and self-identity conflict	Sometimes I’m still unsure whether I’m an ordinary civilian or a veteran, there’s a bit of identity blur. (case 18)
		labeling	People around me think I look sharp after returning from the army, but sometimes I’m also seen as distant. (case 20)
	Lifestyle and routine conflicts	Conflicts in living habits	After coming back, for quite a long time my routines didn’t align with those of my family and friends, and people would wonder whether I was keeping my distance from them. (case 9)
		Different life rhythms	When I had just returned, I couldn’t adjust to the pace of life: I wasn’t in a good state, couldn’t sleep at night, and had no energy during the day. (case 22)
		Differences in consumption concepts	My spending habits were affected… At first after returning, I still kept the consumption habits from the army. (case 22)
		Diverse hygiene practices	I continued to keep certain hygiene practices and daily routines; unlike my classmates, I felt that what they considered “tidy” was not tidy enough. (case 17)
	Decline in proactive behavior	Low motivation for proactive socializing	I feel there are things I just can’t say to my classmates; I still prefer chatting with classmates who have served in the military. (case 17)
		Weak sense of participation	Apart from handling paperwork, I have almost no day-to-day interaction with the relevant departments. (case 21)
		Diminished social drive	I don’t really want to take the initiative to meet people. (case 11)
	Family and psychological gaps	Family’s lack of understanding	Family members hope that after you come back you can start working quickly, get arranged into a job, but some veterans may need a period of adjustment. (case 13)
		Emotional fluctuations	In the period after returning, it’s easy to become irritable; small frictions with classmates can magnify emotions. (case 18)
		Great pressure	After coming back, there’s a clear sense that your energy isn’t as good as it was in the army, and the psychological pressure is heavy. (case 8)
		Weak sense of independence	There are procedures, but they’re scattered, and you often don’t know what the next step is. (case 6)
	Difficulties in social integration and belonging	Difficulty integrating into classes	After returning from military service, I switched from the humanities to engineering, and the process wasn’t very smooth. (case 23)
		Dormitory relationship barriers	When I entered the dorm, my roommates looked confused; I felt out of place and just greeted them quietly. (case 17)
		Low peer recognition and lack of belonging	When I had just come back, I felt it was hard to fit into my social circles. (case 22)
	Cultural and environmental adaptation	Maladaptation to local culture	The city suddenly feels unfamiliar; my daily rhythm is kind of “off the rails.” (case 11)
		Communication barriers	When chatting with peers, there are times when I can’t keep up with the pace. (case 17)
		Difficulty adapting to social norms	Military life is blunt and straightforward. Outside, you have to weigh your words when communicating; can’t keep up with peers’ pace, the latest internet memes. (case 12)
	Information and policy barriers	Cumbersome Identity Transition Procedures	Some subsidy mechanisms should still be implemented more concretely. (case 24)
		Non-transparent policy processes	The policies mention preferential treatment, but many procedures still require us to ask on our own, and it’s unclear what benefits are actually available. (case 21)
		Information asymmetry	There was no notice or guidance on how to continue (or transfer) social insurance; this is an area that needs improvement. (case 23)
Protective factor	Active personal adaptation	Proactive remedial study	After coming back, I took the initiative to study and ask questions, teasing out the parts I didn’t understand bit by bit. (case 17)
		Enthusiasm for learning	Others can finish learning it in a day; I often need a day and a half, but as long as I make up for it little by little, I keep moving forward. (case 19)
		Outgoing personality	I made myself change from being shy and introverted to being cheerful and outgoing. (case 22)
		Optimistic mindset	I think after coming back I’ll go out every week, hoping to see the mountains and waters. (case 16)
	Life skills enhancement	Improved practical ability	There aren’t any ready-made rules or routines, so you have to figure everything out by doing it yourself. (case 8)
		Enhanced life skills	We save on printer paper whenever we can, and since coming back, when we eat out we also pack up the leftovers whenever possible. (case 16)
	Self-directed goal orientation	Proactive future planning	I will put away (save) the resettlement funds provided by the army and the local authorities; I have a plan and won’t spend lavishly. (case 22)
		Setting new goals	As for goal-setting, I have set new goals for myself, I have goals. (case 25)
		Increased self-confidence	We veterans take part in trainings to learn more new knowledge, which makes us more confident in our work. (case 8)
	Family emotional support	Family care	My family has given me a lot of encouragement and help. (case 25)
		Parental tolerance	My family says they will support me no matter what I choose. (case 16)
		Financial security from family	Another thing is the support from my family; in terms of money and cash flow, they have certainly provided most of it. (case 11)
	Comrade support	Sharing experiences among veterans	When I had just returned and was adapting to local life, my comrades gave me a lot of experience-based support. (case 14)
		Mutual assistance among returnees and support from veteran associations	In the Camouflage Association, those who returned in the same year are more familiar with one another; everyone is very warm and we help each other. (case 16)
	Mentorship and guidance	Proactive engagement from mentors	Teachers will tell you about exams, postgraduate entrance exams, certification tests, and joining the Party, and remind you of things to watch out for. (case 17)
		Guidance from counselors	The counselor would often ask us to drop by the office for tea… helping us put down the “soldier’s burden.” (case 17)
		Sharing of experience by senior students	The student veterans on campus have formed a mutual-help setup where the experienced guide the newcomers. (case 16)
	Peer relationship integration	Friendly new classmates	Most classmates were very friendly. That was my strongest impression when I came back. (case 16)
		Proactive help from peers	If someone is hospitalized, everyone chips in to visit and offers comfort and encouragement. (case 21)
		Encouragement from friends	As for my friends, they truly supported me unconditionally, once I made a decision, they backed it. (case 2)
	Organizational and policy empowerment	Exclusive groups	The community keeps records of your demobilization… and there are periodic dedicated work opportunities of that kind, which have been posted in the group. (case 20)
		Bonus policies	There are bonus-point policies for postgraduate entrance exams, and special civil-service positions specifically for veterans in the government recruitment… The eligibility requirements explicitly state the preferential treatment available to veterans. This policy is ongoing. (case 17)
		Information delivery	Staff members regularly post certain information in the dedicated group. (case 1)
		Special subsidies	With so many people leaving the service every year, we also have some job-seeking and entrepreneurship subsidies; and when we look for jobs, companies can be granted tax exemptions/relief because of us. (case 18)
**Stage 3: Readaptation and Reshaping**			
Risk factor	Continued academic pressure	Remedial class pressure	After switching majors we also had to make up the first-year courses, and at that time our sophomore schedule was almost full from Monday to Friday, sometimes even on Saturdays. (case 18)
		Exam anxiety	I failed the postgraduate entrance exam, so I asked my family whether I should try for it again or switch to the civil-service exam. (case 16)
		Lack of academic self-confidence	After such a long time without systematic, formal study, I find the coursework quite demanding. (case 19)
	Skills transfer barriers	Difficulty transferring military experience	The military skills I learned aren’t really usable after discharge. It’s an ongoing issue. Unless you come back and join the police, SWAT, or firefighting. If you had special-operations experience in the service, applying to SWAT can skip the written exam and go straight to an interview. (Case 21)
		Some skills not recognized	For the “second classroom” credits across different majors, it feels like the rules aren’t aligned among the college, the Armed Forces Office, and other departments. Sometimes our service experience runs into problems when being converted into second-classroom credits. (Case 22)
		Limited knowledge transition	That textbook was already more than halfway through, so I had to catch up from earlier chapters. On top of that, the curriculum and training plan changed, so my study time has been consistently tight. (Case 18)
	Identity transition pains	Pressure of adapting to student, soldier, volunteer role transitions	To integrate into the environment, I need to set aside my past identity as a soldier and accept society’s standards. I must overcome my own mindset issues and adapt to my multiple roles, student, volunteer, and also a veteran, gradually accepting the shortcomings I see. (Case 17)
		Role conflict	We veterans tend to be straightforward. That’s an advantage locally, but it can also make it easy to clash with people in everyday interactions. (Case 1)
	Emotional adjustment challenges	Slow emotional recovery	I often dream about re-enlisting. I really miss life in the army and the way things were done there, but there’s no going back. (Case 22)
		Occasional frustration and pressure	I still feel a bit discouraged when facing difficulties. (Case 25)
		Psychological fluctuations	My psychological stress fluctuates from time to time. (Case 18)
	Residual behavioral inertia	Old routines or habits affecting current social and daily life	I eat quickly and often finish before others; it makes me feel out of place. (Case 17)
	Incomplete social boundaries breakthrough	Solidified social circles	Two years might not be enough, because it’s hard to break out of the social circle of one’s family. (Case 7)
		Limited proactive interaction	They won’t take the initiative to contact us, and we don’t take the initiative to contact them either. (Case 16)
		Peer group barriers	I’m about 2 years older than my classmates and our experiences are different—this gap isn’t something that can be closed in a short time. (Case 17)
	Group belonging fluctuations	Unstable sense of belonging	I feel I’m gradually blending into local ways of life, but in my heart I still miss the military. (Case 23)
		Transformation of group identity	In daily life I should set aside the soldier identity, but when responsibility calls I need to step forward. (Case 9)
		Poor class integration	I feel some things just don’t flow when talking with classmates in my major; I still prefer chatting with those who have served. (Case 17)
	Insufficient resource connection	Low utilization of policies and resources	I’ve been there. But the service station rarely organizes activities for veterans, and most of the staff are part-time, so they may not be able to keep up. (Case 14)
		Poor social resource linkage	At the societal level, it mostly depends on veterans themselves to reach out and connect with resources. First, social organizations and groups often don’t know which veterans need help… Second, these organizations may have limited capacity and may not be able to offer adequate support. (Case 1)
		Limited information channels	It’s hard to find the relevant school groups on my own. You mostly get added if you know someone in the group or if your counselor reports your information and you’re then invited. If the counselor doesn’t know about the group, a veteran may have trouble joining. (Case 17)
Protective factor	Personal confidence growth	Restoration of self-confidence	At the village committee we first pooled money to plant oil-tea; when the first harvest turned out well, people gradually regained confidence. (case 8)
		Emotional stability	I’ve made progress in interpersonal communication and can handle problems and manage my emotions more effectively. (case 20)
		Enhanced autonomy	or those who want to start a business… and for those choosing self-employment, we provide skills training to help them find a suitable job. (case 1)
	Skills and abilities enhancement	Growth in practical ability	An older veteran led us through equipment upgrades, even assembling computers. Our hands-on ability became strong. (case 16)
		Learning new social skills	In the army, interaction was simple and direct; outside, communication requires weighing your tone. (case 17)
		Acquisition of life skills	We dug some vegetables from the field and cooked by ourselves to host guests. (case 5)
	Identity upgrade	Recognition of multiple identities	This job gave me a sense of achievement and helped me find my value and direction. Now I’m not only a cadre but also a member of the village. This belonging makes me feel grounded. (case 7)
		Adaptation to new roles	I actively moved away from my previous mindset and clarified what I needed to learn so I could keep up with the school’s pace. (case 17)
		Increased social participation	Among ten freshman veterans, nine joined the college’s routine nucleic-acid testing volunteer service. (case 17)
	Behavioral self-remodeling	New routine formation	I keep regular hours… first maintain an optimistic attitude, face things positively, and better mesh with people around me. (case 24)
		Upgraded lifestyle	With improved finances, I had new needs to purchase household and personal items. (case 17)
		Gradually normalized daily behaviors	I began to participate in village work and slowly found a new direction. (case 3)
	Family emotional support	Deeper family understanding	Teachers… and family… especially between spouses, without that support, the harm to veterans can be great. (case 1)
		Encouragement from family	My family said they would always support me no matter what I choose, we’re an open-minded family. (case 16)
	Mentorship and peer support	Guidance from teachers	Teachers provided guidance on academic policies… informed us of news and reminded us of key matters. (case 17)
		Support from senior students	Teachers and seniors who had served in the military could offer support and suggestions. (case 17)
		Mutual help from classmates	Classmates were very enthusiastic, we helped each other. (case 18)
	Linking social resources	Smooth policy connections	After the teacher coordinated things and with classmates’ help, we could make up the missed courses. It worked out pretty well.” (case 19)
		Exclusive group notifications	There’s a dedicated group that regularly posts job information. (case 10)
		Support from social organizations	The community has a veterans’ service station (the interaction varies by person). (case 17)
	Establishment of new relationships	Participation in class activities	The campus Camouflage Association organizes activities, with funding from the Armed Forces Office. (case 14)
		Joining organizations	The campus Camouflage Association organizes activities, with funding from the Armed Forces Office. (case 14)
		Expanding new social circles	In the library I saw someone wearing the same training T-shirt; I greeted him, and we gradually became familiar. (case 18)
		Volunteering experience	I volunteered to help with high-school military training; seeing the kids progress step by step made me feel truly needed. (case 22)
**Stage 4: Resilience Leap**			
Risk factor	Fluctuations in growth motivation	Insufficient sense of achievement	At the moment I haven’t achieved much of anything. (case11)
		Intermittent motivation	After I came back, I didn’t maintain things well; I kind of reverted to how I was before the army, back to that loose, undisciplined state. (case7)
		Vague stage goals	When it comes to setting goals, I do have goals, but they’re not clearly defined. (case20)
	Barriers to ability breakthrough	Bottlenecks in organizational, management, and learning ability	My learning ability has declined. Secondly, I need to improve capabilities and qualities. (case1)
		Insufficient leadership	I’m a bit lacking in moral or spiritual leadership, in leadership generally. It often makes people feel they can’t really fit in. (case7)
	Limited applicability of experience	Incomplete transfer of experience in new environments	The skills from the military may not be applicable in civilian settings. (case 17)
		Gaps between professional skills and social needs	Military skills… can’t be used. (case21)
	Emerging role pressure	Multiple identities and roles, increasing sense of responsibility but also increased pressure	Having enjoyed so many… benefits and rights… we should also fulfill our obligations. (case 5)
	Mood fluctuations	Occasional mood swings	I’ve been back quite a while and no longer feel the initial discomfort, but when I run into difficulties I still get discouraged easily and lack sustained motivation for growth. (case25)
		Setbacks in self-confidence	Sometimes I feel society doesn’t recognize veterans’ contributions, which undermines my confidence. (case21)
		Stress under frustration	When I fail, I tend to negate myself and need much stronger psychological self-regulation. (case8)
	Life rhythm challenges	Conflicts in time management for work, study, and life	The curriculum and training plan changed, so study time is quite tight. (case 20)
	Intensified resource competition	Limited platform resources	First, perhaps there aren’t enough platforms… we need to build platforms to bring in government and grassroots social organizations… (case10)
		Few entrepreneurial opportunities	For employment support and entrepreneurship programs, they require operating for over 6 months with an actual project… but many veterans need funding right now. (case1)
		Fierce competition for social support	It’s relatively hard to join the Party after demobilization… the college told us not to compete with other students; our group would run through another track. (case16)
	Delayed feedback	Limited social recognition and weak achievement feedback	Previously… I ran an e-commerce store, but it didn’t help much; I couldn’t extend the promotion to farther places. (case11)
		Untimely positive reinforcement	Policy publicity is extensive, but the tangible gains for individual growth feel average. (case 21)
Protective factor	Continuous ability growth	Improved organizational and leadership skills	You have to rally villagers, nothing gets done by fighting alone. (case 16)
		Active learning	At the beginning I learned from experienced colleagues and asked whenever I didn’t understand… over the years I’ve become quite proficient. (case 3)
		Enhanced practical abilities	Engineering management, conflict mediation… flood and drought control—most key tasks were assigned to me, and I completed them. (case 6)
	Goal-oriented planning	Clear goal setting	I’m someone with very clear goals. (case 4)
		proactive future planning	A village needs a solid plan for development… without one you can’t proceed. You plan in advance and then move forward step by step. (case 15)
		Continuous self-reflection	Think more, observe more, study more, listen more, learn from others’ experience and make it my own. (case 15)
	Positive and resilient attitude	Optimistic persistence	When things happen, I won’t give up easily. I’m stronger, more decisive, and more optimistic. (case 2)
		Honor-driven motivation	Many veterans join volunteer work to give back to society, driven by a soldier’s sense of duty. (case 10)
		Mature emotion management	My emotions are fairly stable… in interactions I manage my emotions well. (case 4)
		Consolidated self-confidence	My perseverance has made a qualitative leap… when trouble comes, don’t panic, heaven won’t fall. (case 2)
	Internalization of social responsibility	Active participation in volunteer services	Veterans took part in volunteer service. There were news reports and commendations. (case 12)
		Social contribution	Helped poor households out of poverty and improved infrastructure, this gave me belonging and purpose. (case 15)
		Sharing experiences with new members	Comrades passed on their experience in running a farm. (case 14)
	Optimized lifestyle	Healthy routines and good living habits	The army’s daily routine, reinforced during militia training, reminds me not to forget my responsibilities. (case 6)
		Frugality and self-discipline	I saved the demobilization payment to plan my studies and life. (case 6)
	Family and social support	Continuous encouragement from family	My wife runs the household so I don’t have to worry… leaders value me more… friends support me unconditionally. (case 8)
		Expanding social networks	I’m in multiple groups; when something comes up people will help if I call; we also gather during holidays. (case 5)
		Mutual support from comrades	When facing difficulties, if the other person is also a veteran we work together. There’s a natural mutual help and identity bond. (case 5)
	Organizational and policy empowerment	Teamwork	Stronger collective consciousness and we do what we promise. (case 17)
		Policy incentives and employment services	Support and training from the university and the Veterans Affairs Department. (case 20)
		Entrepreneurial support	Policies for veterans’ entrepreneurship training/loans/skills training… the state provides strong support. (case 11)
	Resource integration and innovation	Effective use of alumni, policy and social resources	Leveraged related policies and funds to complete the village plan. (case 15)
		Interdisciplinary communication	We visited many markets, connected with e-commerce platforms and large wholesalers, and stabilized sales channels. (case 15)
		Innovation and entrepreneurship	Started a farm after demobilization, integrating agriculture and tourism. (case 14)
**Stage 5: Societal Reintegration**			
Risk factor	Pressure of identity integration	Pressure of switching between multiple identities	You have to integrate into the environment, let go of the former identity. (case 17)
		Gap in social expectations	Veterans must work to adapt to society and make changes themselves—don’t assume serving means you’re inherently extraordinary. (case 12)
		Difficulties in balancing family and social roles	If there’s a stable job near home, just being able to look after my wife and kids is enough. (case 19)
	Contribution value anxiety	Limited social feedback and insufficient positive feedback	Have you interacted with local community organizations? No… There isn’t a way to get in touch, no channel to connect. (case 18)
		Weakened sense of contribution	I can’t really talk about “contributions”… (case 10)
	Challenges in capacity expansion	Difficulties in adapting to new domains	It wasn’t smooth. (case 23)
		Bottlenecks in integrating cross-sectoral resources	These aren’t completed, even facial-recognition access won’t work. (case 21)
		Challenges in further education or innovation	There are many challenges ahead, and I’m not sure I can overcome them all. (case 16)
	Fluctuations in sense of wellbeing	Fluctuations in family happiness, life satisfaction affected by social pressure	Without support from leaders, colleagues and especially one’s spouse, the harm to a veteran can be significant. (case 1)
	Obstacles to experience transfer	Difficulty in effectively passing on experiences to new groups	Early on there were many difficulties: villagers didn’t trust the cooperative and wouldn’t join… some young people wouldn’t stay in the countryside, thinking agriculture has no future. (case 7)
		Low motivation for policy advocacy	
	Recognition and respect dilemma	Lack of social recognition, weakened sense of honor	At school you may face classmates’ lack of understanding and uncooperative staff… In the wider community you may encounter misunderstanding or ridicule. (case 17)
		Being misunderstood or marginalized	I attended a county Veterans Affairs meeting in 2021 as a rural veteran representative. Frankly, after returning from the military, people tend to relax; they just want a break. They don’t really have the mindset to attend employment training, some do go, but not many. (case 4)
	Service and responsibility pressure	Burden of regular social service and public welfare	Carrying out many tasks even requires us to pay out of pocket. (case 5)
		Increased entrepreneurial pressure	At first, we weren’t familiar with the market; when our oranges and peppers first came out, we even faced unsold stock. (case 7)
	Barriers to social network expansion	Difficulties in expanding cross-sectoral networks	At the societal level, it mostly depends on veterans themselves to reach out and connect to resources. (case 1)
		Unstable social support systems	The veteran privilege card can only be used within a given region… since my long-term destination isn’t set, I haven’t applied. (case 19)
Protective factor	Integration of multiple identities	Rebuilding self-confidence	Since I joined the village committee in 2018, I’ve received county- and city-level awards almost every year. That is the government’s recognition of our work, and these results spur us to work even harder. (case 4)
		Flexible identity switching	To blend in, I had to set aside the soldier identity and accept social norms, work through my own mindset, gradually accept shortcomings I see, with guidance from teachers, classmates, and family elders. (case 17)
		Balancing family and social roles	I feel fortunate to have an open-minded family. (case 16)
	Experience sharing and motivation	Giving back to new members	When younger students ask about enlisting, I answer warmly and share what I know. (case 16)
		Active policy advocacy	When villagers face practical difficulties they can’t solve, we actively report to the relevant local departments, coordinate, and resolve them promptly. (case 10)
		Positive encouragement within the group	By taking part in activities, we contributed to the school and students; that passion and sense of service earned praise. (case 17)
	Mature lifestyle	Awareness of healthy living	They guided us toward healthy, hygienic living, how to manage life and value education. (case 10)
		Self-discipline	How did I adjust? I mostly didn’t—just kept my own schedule…and held myself to strict standards. (case22)
		Stable family happiness	My family is very supportive. (case 2)
	Mature and stable mindset	Mature emotion management	Now I look at things more comprehensively, not just thinking of myself but about how my actions affect others… If there’s a dorm conflict, stay calm and avoid impulsiveness.” (case18)
		Strengthened sense of responsibility	I’m quite responsible at work and always see things through. (case6)
		Sustained goal planning	I set aside the resettlement funds from the military and locality, planning my finances instead of spending loosely. (case 22)
	Family and social support	Encouragement from family	my family. There’s a lot of encouragement and help from them. (case 25)
		Mutual help from peers	I’m in multiple groups, call out and people will help. (case 17)
		Coordinated social resources	After the training, we polled in the group to find a time and then organized small, targeted job fairs for those needing work. (case 12)
	Social recognition and achievement	Gaining social respect	Both my personal and social value have been fully recognized. (case 9)
		Honor-driven growth	By contributing to the college and students through activities, I received praise and encouragement from the dean. (case 17)
		Increased sense of happiness	In recent years our village’s living standards have improved a lot. That’s what gives me the strongest sense of achievement. (case 8)
	Normalization of social service	Internalization of public service	Watching the kids progress step by step made me feel I was doing something significant. (case 22)
		Social contribution	As for contribution, I’ve stayed committed from the beginning up to now. (case 12)
		Increased motivation for innovation and entrepreneurship	Many young people are returning to start businesses, our village is getting more vibrant. (case 7)
	Expansion of circles and abilities	Enhanced cross-sectoral social skills	It mainly improved my interpersonal skills, whether dealing with sub-district offices or others, it’s all communication. (case 11)
		Enriched social networks	A few of us veterans still keep in frequent contact. (case 2)
		Continued development	I feel my ability to handle these matters is continuously improving. (case 22)

### 3.3 Selective coding and theoretical integration of the dynamic mechanism of veterans’ resilience

Based on the results derived from open and axial coding of veterans’ resilience across its dynamic stages, together with the associated risk and protective factors at each phase, a clear pattern of continuous evolution emerges, characterized by a dynamic interplay of reduction, transformation, and regeneration. Throughout these phases, risk and protection do not function as static entities but as interwoven and dialectical forces. During the initial stage of identity rupture, the sense of disorientation and identity fragmentation is partially alleviated by emotional support from family members and comrades. In the subsequent re-adaptation phase, the pressures of environmental and role adjustments are mitigated through the gradual emergence of new social belonging and acceptance. As veterans enter the growth transition phase, anxiety surrounding competition and self-expectations coexists with the progressive development of competencies and goal orientation, ultimately culminating in the advanced integration of personal identity and societal contribution. This dynamic trajectory highlights how risk factors act as catalysts for behavioral adaptation, compelling veterans to engage in coping strategies, while protective factors reinforce resilience and foster upward developmental momentum. These factors operate within a continuous feedback loop in which each exerts influence on the other. The selective coding of this dynamic mechanism is presented in Table 5, which delineates a five-stage framework grounded in the core trajectory of risk and protection. This structure reflects the transitions and evolving relationships between risk exposure and protective reinforcement across the stages of resilience development.

**TABLE 5:** Selective coding of the dynamic mechanism of veterans’ resilience.

Resilience stage	Core risk axis	Core protective axis	Main dynamic and interaction logic	Primary mechanism of action
Identity Separation	Identity and belonging disruption; skills and resource barriers	Family, comrade, peers and campus support; personal will and life skills	Risks emerge from identity rupture and resource gaps, buffered by close social ties and internal capacities	Risk-dominant, protection as buffer
Adaptation Conflict	Role and identity conflict; cultural and integration barriers	Active personal adaptation; peer, mentor, and policy support	Risks stem from adaptation difficulties, while protection derives from individual strategies and external empowerment	Stress and adaptation alternating
Readaptation and Reshaping	Academic and social pressure; fluctuation in resources and belonging	Confidence development; relationship rebuilding; social integration	Ongoing adaptation creates pressure, which is transformed into growth momentum through rising self-efficacy and enriched social resources	Stress transformed into growth momentum
Resilience Leap	Motivation fluctuation; resource competition; role pressure	Skill development; goal setting; positive mindset; internalized responsibility	Risks emerge from new challenges and competitiveness, while protection is sustained by growth motivation and dual empowerment (internal and external)	Risk stimulates growth, protection facilitates
Societal Reintegration	Identity integration pressure; anxiety over recognition and contribution; network barriers	Integration of multiple identities; social achievement; normalized service and support	Risks stem from reintegration barriers, while protection arises from stable self-concept, social support, role mastery, and value realization	Advanced integration and social contribution

**FIGURE 1:**
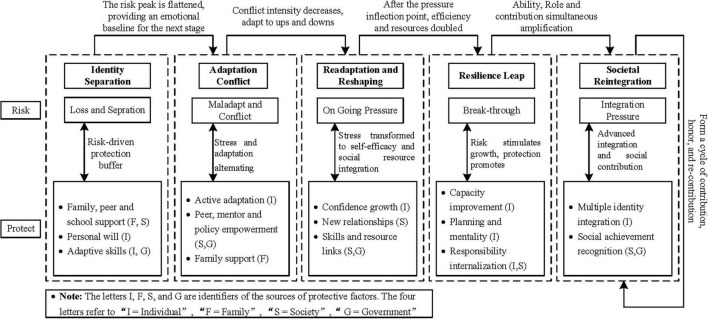
Dynamic mechanism model of vetrans’ resilience.

Anchored in the five-stage evolutionary framework, this model systematically integrates both risk and protective factors, along with their respective sources, into a coherent temporal trajectory of veterans’ resilience development. By delineating the specific triggering mechanisms that initiate each stage and the transformation mechanisms that drive progression to subsequent stages, the model captures the complex and dynamic interplay between vulnerability and adaptability throughout the resilience-building process. This analytical structure provides a comprehensive representation of how veterans navigate transitions from initial disruption through phases of adaptation, recovery, and growth, culminating in a state of advanced integration defined by identity reconstruction, social reintegration, and value contribution. The resulting dynamic mechanism diagram visually illustrates this transition from a predominantly risk-oriented phase to a mature stage of resilient integration, offering a theoretically grounded and empirically informed depiction of the resilience trajectory in the veteran population, as depicted in Figure 1.

Here we present two typical life history narratives that illustrate the developmental linkage and transformation mechanisms across the five phases which are Identity Separation, Adaptation Conflict, Readaptation and Reshaping, Resilience Leap, Societal Reintegration of veterans’ resilience.

Case 8. Following demobilization, he entered the Identity Separation stage in which an abrupt rupture of role identity and career direction left him briefly considering outside employment before local persuasion led him to remain and serve his village. During the subsequent Adaptation Conflict stage his early reinsertion revealed gaps in legitimacy and procedural norms and limited trust from villagers, creating situational strain and pressures on his credibility. Supported by family and comrades and through sustained immersion in daily village affairs he progressed into the Readaptation and Reshaping stage, rebuilding routines and cultivating a situated sense of belonging. A clear Resilience Leap occurred when the village committee collectively financed a pilot camellia project; the project produced visible yields and mobilized external support, which together converted local skepticism into a shared sense of efficacy. Over successive seasons camellia revenues broadened participation and improved living standards, and his personal achievement became closely linked to community welfare, marking Societal Reintegration in which identity reconstruction, external recognition, and place-based value contribution converged into a stable social position. Taken together this life history exemplifies a reduction, transformation, and regeneration dynamic in which early disorientation and distrust catalyze adaptive problem solving that, when reinforced by protective resources, culminates in durable and socially validated confidence.

Case 9. On returning to civilian life, he underwent Identity Separation as the abrupt loss of military routines undermined daily anchors of identity and required a reconfiguration of personal purpose. Entering the Adaptation Conflict stage, his move into agricultural entrepreneurship confronted chain closure and marketization challenges that demanded both successful production and successful sales, thereby intensifying capability environment frictions. He then adopted a periodized strategy that constituted the Readaptation and Reshaping stage by framing a difficult 3–5 years startup period followed by a 5–10 years development cycle; this temporal structuring stabilized goals and provided a framework for competency consolidation. By leveraging institutional linkages such as interest subsidized financing and targeted policy programs he initiated a Resilience Leap that compressed transactional frictions and converted uncertainty into growth momentum. Accumulating recognition allowed him to diffuse disciplined, military honed execution norms into enterprise practice and to assume a teaching role at the district Party school for successive cohorts of veterans, reflecting Societal Reintegration in which confidence is grounded in system level endorsement rather than solitary struggle. This trajectory traces the full five stage mechanism and demonstrates how iterative interactions between risk exposure and protective reinforcement progressively reconfigure vulnerability into a stable identity of contribution and leadership.

### 3.4 Assessment of saturation

We assessed conceptual coverage by conducting a theoretical saturation exercise aligned with grounded theory procedures. After performing open and axial coding on 25 interviews to develop the codebook and the preliminary model, we constructed a saturation grid that mapped all emergent initial concepts, categories and inter-category relationships to specific cases. Applying the constant comparison method, we then coded an additional set of 12 interviews held aside for saturation testing, resolved coding discrepancies through consensus meetings and preserved a complete audit trail. For case selection the research team actively sought and examined negative and deviant cases via theoretical sampling to test emerging propositions and probe boundary conditions, and these cases were explicitly compared with the developing paradigm linkages. Across the saturation set no new initial concepts emerged, no existing categories required modification or further subdivision and no novel relationships between categories were identified, with all data accommodated within the established coding framework. Analytic memos recorded increasing conceptual density and redundancy and our a priori stopping criterion of two consecutive interviews yielding no additional codes or links was met within the saturation sample, indicating that thematic saturation at the group level had been achieved.

## 4 Discussion

### 4.1 Dynamic structure of risk and protection within veterans’ resilience mechanisms

The resilience of veterans during their reintegration into civilian society is not a linear phenomenon but a complex and dynamic process that evolves across multiple stages. Drawing on grounded theory analysis and a five-stage coding model comprising Identity Separation, Adaptation Conflict, Readaptation and Reshaping, Resilience Leap, and Societal Reintegration, this study constructs a theoretical framework that explains how risk factors and protective factors interact within each stage and collectively shape the trajectory of veterans’ resilience.

In the Identity Separation stage, veterans often experience an abrupt break from military norms that causes emotional instability, role ambiguity, and disconnection from institutions. “When I suddenly lost contact with my comrades in arms, I felt lost and unsettled” (case 23). “Suddenly, my goals don’t feel so clear anymore” (case 7). “When I was about to be discharged, just thinking about what I could do back home got me all worked up; I didn’t know what kind of job I could do” (case 9). “The skills learned in the army are all military skills, which are basically not usable in study or work” (case 21). These risks undermine a veteran’s sense of belonging and routine competence. They also make it hard to navigate civilian institutions when administrative procedures and campus rhythms no longer match prior military experience. Protective resources reduce these threats through close relationships and targeted supports. “Overall, it’s the family that has offered more care and also some financial support” (case 11). “With former comrades, we used to talk a lot while serving; after returning to our local communities, we keep communicating and exchanging with each other” (case 4). “A veteran who had been discharged before me rode his bike and took me here and there; although he was a stranger, he made me feel very warm” (case 15). In sum, the stage is risk-dominant: identity disruption and resource gaps drive the process, while family support, comradeship, and campus networks act as stabilizing buffers that prevent psychological breakdown and help veterans move gradually toward later reintegration phases.

The Adaptation Conflict stage appears when veterans face clear role dissonance and trouble readjusting everyday routines. It builds on the ruptured sense of belonging and the procedural opacity seen in the Identity Separation stage. In this phase, friction grows between the rigidity of military discipline and the flexibility required by civilian life. Stress comes from identity uncertainty, mismatched daily rhythms, and cultural barriers. At the same time, protection increasingly comes from intentional strategies and social supports that help gradual reintegration. “To fit in, I need to set aside my former identity as a soldier and accept society’s standards” (case 17). “Sometimes I’m still unsure whether I’m an ordinary civilian or a veteran, there’s a bit of identity blur” (case 18). These accounts show how ambiguous role positioning and unclear labels create psychological strain. “After coming back, for quite a long time my routines didn’t align with those of my family and friends, and people would wonder whether I was keeping my distance from them” (case 9). This example demonstrates how different daily rhythms and habits can block peer integration. Veterans also adopt deliberate learning and use social resources to manage tensions. “Others can finish learning it in a day; I often need a day and a half, but as long as I make up for it little by little, I keep moving forward” (case 19). “In the Camouflage Association, those who returned in the same year are more familiar with one another; everyone is very warm and we help each other” (case 16). “We veterans take part in trainings to learn more new knowledge, which makes us more confident in our work” (case 8). These examples illustrate proactive skill remediation, mutual assistance, and targeted training that increase self-efficacy and turn procedural help and information into steady progress despite ongoing role conflict and cultural adaptation pressures.

In the Readaptation and Reshaping stage, veterans still face structural pressures while internal dynamics begin to shift as adaptive processes take hold. Persistent academic burdens manifest as having to retake foundational courses with an overloaded timetable so that the “sophomore schedule was almost full from Monday to Friday and sometimes even on Saturdays” (case 18). Exam-related uncertainty appears when a veteran reports “failing the postgraduate entrance exam and debates whether to try again or switch to the civil-service exam” (case 16). Skills-transfer barriers remain pronounced for those who find that “military skills are not readily usable after discharge unless they re-enter specific professions such as police or firefighting” (case 21). Despite these risks, protective mechanisms emerge. Collective economic initiatives restored confidence when “pooled village funds produced a successful first harvest and people gradually regained confidence” (case 8). Structured mentorship and peer support from teachers and senior students provided guidance on academic policies and timely reminders that helped veterans navigate institutional requirements. (case 17) Informal hands-on learning strengthened practical abilities when an experienced veteran led equipment upgrades and trainees acquired strong hands-on skills. (case 5) Together, these risk and protective factors show how ongoing external demands interact with growing personal efficacy, social linkage, and role adaptation, converting continual stressors into opportunities for learning and identity reconstruction even though full social integration has not yet been achieved.

The Resilience Leap stage represents a nonlinear acceleration in capacity development and intrinsic motivation. Veterans at this stage translate stabilized routines and growing self-efficacy into proactive, future-oriented initiatives. Risks here arise less from basic adaptation needs and more from fluctuating motivation, capability bottlenecks, and intensified competition for scarce resources. For example, some veterans report an insufficient sense of achievement that undermines sustained effort. One veteran stated, “At the moment I haven’t achieved much of anything” (case11). Others describe vague goal definitions that limit directional progress. A veteran observed, “When it comes to setting goals, I do have goals, but they’re not clearly defined” (case20). Capability barriers further constrain advancement. As one veteran admitted, “My learning ability has declined. Secondly, I need to improve capabilities and qualities” (case1). Resource competition amplifies pressure when platforms and opportunities are perceived as limited. One veteran noted, “First, perhaps there aren’t enough platforms… we need to build platforms to bring in government and grassroots social organizations” (case10). Protective processes counterbalance these risks. Veterans consolidate leadership and practical skills. One shared, “You have to rally the villagers, nothing gets done by fighting alone” (case16). Others embrace active, iterative learning. For example, a veteran said, “At the beginning I learned from experienced colleagues and asked whenever I didn’t understand… over the years I’ve become quite proficient” (case3). Clear goal orientation and honor-driven civic motivation also provide protection. One veteran explained, “I’m someone with very clear goals” (case4). Another added, “Many veterans join volunteer work to give back to society, driven by a soldier’s sense of duty” (case10). Together, these risk and protective patterns signal a shift from mere coping toward structured self-actualization. Targeted support for goal clarity, capability upgrading, and platform access can transform emerging vulnerabilities into durable growth pathways.

In the Societal Reintegration stage, veterans face persistent challenges related to identity reintegration, uneven social recognition, and ongoing service obligations. These pressures become apparent in veterans’ own accounts. Case 17 illustrates identity tension: “You have to integrate into the environment, let go of the former identity.” Case 18 highlights limited social engagement channels: “Have you interacted with local community organizations? No… There isn’t a way to get in touch, no channel to connect.” Case 5 shows material and logistical strains from routine public duties: “Carrying out many tasks even requires us to pay out of pocket.” Protective processes that support resilience emerge through intentional identity reconstruction and institutional recognition. Case 4 captures this: “Since I joined the village committee in 2018, I’ve received county and city level awards almost every year. That is the government’s recognition of our work, and these results spur us to work even harder.” Experiential mentorship and knowledge transfer also play a role, as in case 16: “When younger students ask about enlisting, I answer warmly and share what I know.” Finally, sustained family encouragement provides support, exemplified by case 25: “My family, there’s a lot of encouragement and help from them.” Taken together, these risk and protective factors suggest that resilience at this stage arises not solely from individual adaptation, but from the alignment of multiple social identities, accessible channels for contribution, and tangible recognition. Veterans who experience this alignment report greater wellbeing, more stable social returns, and a consolidated, advanced state of resilience.

### 4.2 Core features of the veterans’ resilience development pathway

Unlike prior studies that primarily corroborate the roles of social support, identity reconstruction, and resource accessibility in veterans’ resilience (e.g., Li et al., 2023; Pietrzak and Cook, 2013), the veterans’ resilience development pathway in this study departed from a purely confirmatory account and reinterprets phase specific functioning across Identity Separation and Societal Reintegration. Building on rigorous empirical investigation and a systematically developed grounded theory approach, we specify how early relational buffering, especially from family, peers, and institutions, mitigates identity detachment during initial disorientation, and how later protective processes shift toward social achievement, relational restoration, and multi-role integration during reintegration. On this empirical and theoretical basis, the study advances existing frameworks by proposing three distinctive mechanisms that refine rather than merely align with prior theory: (1) policy exposure as an identity validating resource, whereby institutional contact confers symbolic recognition that stabilizes role identity beyond instrumental support; (2) peacetime demobilization induced identity rupture within a collectivist cultural context, in which organizational withdrawal and value dissonance, rather than combat trauma, constitute the primary stressors that shape the temporal evolution of protective factors; (3) role backlash among high functioning veterans after reintegration, capturing paradoxical pressures such as anxiety about sustaining contribution, emotional fatigue, and dissonance between achievement and recognition that persist beneath outward success. Together, these mechanisms extend phase-specific resilience theory by clarifying how protective resources transform across transitions and by identifying previously under theorized identity processes in collectivist settings.

Different from traditional general resilience models that conceptualize resilience as a static personality trait or as a linear process of returning to a pre-crisis equilibrium (Luthar et al., 2000; Masten, 2001), resilience among veterans is described as a temporally structured and multi-phase developmental trajectory that is rooted in identity shifts and ecological interactions. A distinctive characteristic of veterans’ resilience lies in its phase specific delineation of risks, progressing from early identity dislocation and social withdrawal, through mid-phase cultural dissonance and role ambiguity, and culminating in late phase pressures associated with social contribution and symbolic recognition. This dynamic risk structure differs from previous models, which often classify adversity as acute or chronic without fully accounting for transitional variability. Protective factors are also presented as evolving across these phases. They begin as relational anchors such as family and comrades, extend into structural scaffolds including institutional mentoring and policy support, and ultimately develop into internalized capacities such as civic engagement and future-oriented planning. Crucially, our findings refine existing theory by showing how policy exposure functions as identity validation: the visibility and accessibility of reintegration policies transform external scaffolds into internalized commitments, positioning adversity as a catalyst for identity expansion and psychosocial growth rather than as a fixed psychological threat. The Societal Reintegration phase thus extends beyond individual adaptation to highlight relational reconstruction, civic reintegration, and pluralistic identity integration. This interpretation foregrounds the policy system’s identity-validating function within the institutional ecology, thereby effecting a substantive extension of existing theory.

Veteran-specific resilience literature, predominantly from American, Canadian, and Israeli contexts, typically defines resilience either as an outcome such as the absence of PTSD or stable employment, or as a latent psychological trait such as grit or emotion regulation (Mobbs and Bonanno, 2018; Pietrzak and Cook, 2013; King et al., 1999). These perspectives are largely derived from post-traumatic models focused on combat-intensive populations. In contrast, this framework reconceptualizes resilience as a longitudinal identity transformation that is shaped by phase-specific psychosocial dynamics. It partially aligns with ecological and adaptive models (Ungar, 2011) but extends them by placing emphasis on reconstruction, integration, and contribution across sociocultural and institutional transitions, which remain underexplored in current theories. Distinctively, in a collectivist cultural context where military belonging is symbolically and institutionally reinforced, peacetime demobilization produces an identity rupture that is not rooted in combat trauma but in the abrupt withdrawal of organizational structures and the fragmentation of shared values. In contrast with trauma-centered models grounded in post-combat experiences (Bonanno et al., 2012; Ackerman, 1945; Mobbs and Bonanno, 2018), Chinese veterans typically demobilize under peacetime conditions. Their psychological distress arises more from institutional detachment and value dissonance than from combat trauma. Resilience is therefore conceptualized as a response to abrupt organizational withdrawal and fragmented sociocultural norms. Veterans face unfamiliar civilian expectations, weakened support infrastructures, and the uncertainties of a market-based society. This framing moves beyond trauma recovery and reflects a process of renegotiating identity and value within a civilian context. Unlike developmental or displacement frameworks, such as those addressing child development or refugee resettlement where identity remains in formation, veterans transition from rigid institutional roles to ambiguous and discontinuous civilian expectations (Demers, 2011; McDaniel et al., 2024). The findings illustrate a dynamic identity trajectory that progresses from rupture to hybrid integration, which is most apparent in the Resilience Leap and Societal Reintegration phases. This trajectory advances role theory by underscoring identity instability, rather than fixed deficits, as a principal mediator of reintegration outcomes.

While prior studies frequently treat reintegration as the final endpoint of resilience (Saleem et al., 2020; Romaniuk et al., 2024), the framework presented here identifies emerging psychosocial pressures among high-functioning veterans. These include anxiety over sustained contribution, emotional fatigue, weakened peer relationships, and dissonance with institutional recognition, which are collectively referred to as role backlash. This concept reflects a paradox in which veterans who appear socially successful may privately experience psychological strain and relational challenges. Our model therefore extends existing theory by introducing role backlash as a late-phase vulnerability that can be precipitated by rapid upward mobility and heightened expectations in civic and occupational arenas. Such post-reintegration vulnerabilities challenge the prevailing assumption that external success equates to inner resilience and reveal the need for ongoing, identity-validating policy exposures that convert achievement into durable belonging. They expand theoretical and policy perspectives on resilience beyond trauma recovery by emphasizing the necessity of sustainable identity validation and enduring support mechanisms.

### 4.3 Areas requiring strengthening within the dynamic development of veterans’ resilience

Government policies for veterans in China are disproportionately concentrated on the initial phases of transition, including Identity Separation and Adaptation Conflict, where risks are most apparent and politically sensitive. These policies primarily involve subsidies, employment guidance, and similar measures. However, mechanisms of support for subsequent phases, including reintegration and identity reconstruction, remain significantly insufficient. This situation reflects the absence of a comprehensive full-cycle support framework and the lack of mechanisms for long-term risk tracking. In the absence of continuous institutional involvement, veterans entering the Resilience Leap and Societal Reintegration phases frequently encounter a shortage of psychological guidance, social empowerment, and recognition of their roles. Consequently, this can result in developmental stagnation, persistent ambiguity in social and personal roles, or even regression into maladaptive coping strategies, despite the fact that early-stage support had previously demonstrated effectiveness.

Across all five stages of resilience development, protective factors are primarily derived from the individual level, including self-discipline, willpower, and personal planning, while the contributions of families, communities, and broader social networks remain comparatively limited. This pattern underscores that external buffering and collective responsibility are relatively insufficient within veterans’ processes of social adaptation. The limited involvement of familial and societal actors not only exacerbates psychological isolation but also contributes to deficiencies in identity validation and social recognition. Over time, veterans may begin to perceive resilience as an individual burden, which can ultimately result in emotional fatigue and relational withdrawal.

Currently, mental health interventions for veterans remain reactionary. Structured psychological education is lacking, particularly in addressing emotional fluctuations and role transitions across the different stages of resilience development. No institutional framework has yet been established to deliver stage-specific psychological services that respond to the diverse needs arising throughout resilience progression. As a result, many veterans, especially during the Resilience Leap phase, are left without structured coping mechanisms for managing contribution pressure, identity ambiguity, or uncertainties regarding social belonging. Over time, this absence of guidance contributes to underdiagnosed psychological distress and inadequate emotional support for growth and sustainable reintegration.

Veteran support structures are fragmented across multiple administrative bodies, including the Veterans Affairs Bureau, Civil Affairs, Education, and Employment departments, without cohesive channels for coordination or information exchange. As veterans move through successive resilience stages, the absence of continuity and interdepartmental data sharing results in repeated eligibility screenings, inconsistent service provision, and diminished overall efficiency and accessibility of intervention measures.

Even after achieving surface-level reintegration, numerous veterans continue to grapple with internal uncertainty, social misalignment, or role fatigue. Current systems seldom monitor or address these late-stage risks. Mechanisms for evaluating long-term role satisfaction or alignment with social contribution expectations remain scarce. In the absence of post-integration follow-up, veterans who outwardly appear well-adjusted may nonetheless encounter role dissonance, unmet emotional needs, or disengagement from civic service. These unaddressed challenges can result in a gradual erosion of resilience and undermine the sustainability of prior support efforts.

### 4.4 Policy implications for strengthening veterans’ resilience

Veteran support policies need to cover the entire resilience cycle. This requires the development of a modular support system designed around five development stages, which are Identity Separation, Adaptation Conflict, readjustment, Resilience Leap, and reintegration. At each stage, policy measures should incorporate tailored psychological services, structured social mentorship networks, and clearly defined reintegration benchmarks. Government agencies are expected to coordinate their actions through a unified digital system that enables continuous risk monitoring and ensures timely transitions of support across stages. Such a full-cycle framework minimizes points of disengagement and maintains the adaptive progression of veterans throughout the reintegration process.

The long-term development of veterans’ resilience depends on the embeddedness and operational effectiveness of grassroots service networks. Local governments are advised to strengthen existing community level veteran service stations and neighborhood committees so that they function as frontline actors in resilience building. These grassroots platforms need to evolve from mere administrative contact points into multi-functional service hubs. Beyond managing formal documentation, these stations should be equipped to provide psychological counseling and emotional support. Incorporating peer veterans who have successfully transitioned into service delivery enhances credibility and allows guidance to be delivered in a relatable manner. Partnerships with local communities and civic organizations can further integrate veterans into broader support networks, thereby alleviating social isolation. Reinforcing grassroots service ecosystems ensures that resilience development remains closely aligned with local conditions and that interventions are sustained through relational trust and continuous engagement in daily community life.

Many resilience breakdowns originate from the absence of anticipatory guidance during the transition from military to civilian life. The Ministry of Veterans Affairs should incorporate structured resilience curricula into pre-discharge training programs. These curricula should include training on stress management, strategies for adaptive role transition, and psychoeducation focused on emotional regulation. The programs should be tailored according to service duration, specific military position, and anticipated civilian role. Providing cognitive behavioral training and workshops on narrative identity reconstruction prior to discharge can alleviate emotional turbulence and establish realistic psychological expectations for civilian life.

Fragmented access to services remains a significant barrier, but China’s ongoing integrated e-government reforms offer a feasible pathway for policy expansion. In line with the State Council’s 2018 “*Guiding Opinions on Accelerating the National Integrated Online Government Service Platform*” (《国务院关于加快建设全国一体化政务服务平台的指导意见》), veteran-related resources can be gradually federated, rather than immediately centralized, through existing “one-stop” government portals that support cross departmental data sharing and workflow collaboration, including unified identity/authentication, electronic certificates, and data exchange. In practice, several localities are already piloting veteran focused “one-stop” service packages within provincial or municipal portals, demonstrating a scalable pathway from localized pilots to broader implementation based on local readiness. Instead of mandating rigid biannual checks or creating an entirely new cross departmental system, we propose a staged integration approach: (1) map veteran service items already required under the “*2018 Employment and Entrepreneurship Support Opinions*” (《关于促进新时代退役军人就业创业工作的意见（2018）》), including training, educational benefits, and job-matching, into existing portals; (2) introduce optional self-assessment modules aligned with the five stages resilience framework; and (3) leverage analytics on service utilization patterns to identify early risk signals for local follow up, consistent with the e-government principles of process traceability and data sharing.

Evidence from China’s current veteran policy mix indicates an expanding grassroots service infrastructure and increasing attention to health and psychological support. According to the Ministry of Veterans Affairs’ task briefs (2019) and provincial “Service Center/Station Construction Guides,” counties, townships, and communities are establishing standardized service stations capable of routine outreach, satisfaction surveys, and referrals functions that naturally facilitate lightweight, periodic follow-ups without requiring a new national mandate. Based on such documents, we recommend piloting confidential role satisfaction surveys, employer and family feedback channels, and targeted mental health consultations embedded within existing stations. These measures should be iteratively refined using real world feedback. Institutionalized yet proportionate monitoring can provide continuous, locally adapted support and reduce the risk of late-stage vulnerabilities being overlooked.

## 5 Limitations

This study presents a comprehensive and process-oriented model of resilience development among veterans, grounded in qualitative data and informed by grounded theory. Several limitations should be noted to contextualize the scope and applicability of the findings. Although the five-stage resilience model was rigorously derived through extensive coding of interview transcripts and institutional documents, it remains largely interpretive. The framework enhances conceptual clarity, yet quantitative validation is required to determine the relative effects of various risk and protective factors at each stage. Future research may employ longitudinal survey data or mixed-methods approaches to test causal relationships and confirm the temporal sequencing outlined in this study.

While the model provides a structured understanding of stage-specific resilience dynamics, it does not currently include real time monitoring or assessment tools for practical use. Future work could focus on developing resilience stage indices or digital intervention systems to help veterans and policymakers identify vulnerabilities and act proactively. Further investigation into how institutional and social ecosystems can be systematically aligned with stage-specific needs remains essential for achieving translational impact.

## 6 Conclusion

This research contributes to the expanding literature on veteran reintegration and resilience by constructing a dynamic, stage-sensitive model tailored to the post service context. It demonstrates that veterans’ resilience is neither a static trait nor a mere return to pre-disruption functioning, but rather a long-term transformation driven by identity, characterized by fluid risks, shifting support structures, and evolving personal and institutional demands. The proposed five-phase model, which spans Identity Separation, Adaptation Conflict, Resocialization, Resilience Leap, and Societal Reintegration, provides an explanatory framework that integrates psychological, social, and policy dimensions.

The study emphasizes the misalignment between concentrated early-stage policy support and the emergence of risks in later stages, revealing institutional deficiencies in sustaining resilience cultivation. It further identifies the imbalance between individual-centered resilience efforts and the insufficient development of community and family support systems. By connecting these findings with tangible policy shortcomings such as fragmented inter-agency coordination and the lack of consistent post-integration feedback, the study outlines actionable directions for system level reform. Overall, this research not only advances theoretical understanding of the complex resilience trajectories of veterans in peacetime but also lays a solid foundation for targeted interventions, innovative policy design, and comparative international studies.

## Data Availability

The original contributions presented in the study are included in the article/supplementary material, further inquiries can be directed to the corresponding author.
